# Sexual Functioning, Satisfaction, and Well-Being Among Contraceptive Users: A Three-Month Assessment From the HER Salt Lake Contraceptive Initiative

**DOI:** 10.1080/00224499.2021.1873225

**Published:** 2021-02-09

**Authors:** Jenny A. Higgins, Renee D. Kramer, Kelsey Q. Wright, Bethany Everett, David K. Turok, Jessica N. Sanders

**Affiliations:** a Departments of Obstetrics and Gynecology and Gender and Women’s Studies, University of Wisconsin; b Department of Population Health Sciences, University of Wisconsin; c Department of Sociology, University of Wisconsin; d Department of Sociology, University of Utah; e Department of Obstetrics and Gynecology, University of Utah

## Abstract

Few large, longitudinal studies document multiple contraceptive methods’ effects on sexual functioning, satisfaction, and well-being. We leveraged data from the HER Salt Lake Contraceptive Initiative, a prospective cohort study with patient surveys at baseline, one month, and three months. Surveys assessed bleeding changes, contraceptive-related side effects, sexual functioning and satisfaction, and perceptions of methods’ impact on sexual well-being. Individuals in the final sample (*N =* 2,157) initiated either combined oral contraceptives, levonorgestrel intrauterine devices (IUDs), copper IUDs, implants, injectables, or vaginal rings. Across methods, participants exhibited minimal changes in sexual function (Female Sexual Function Index-6 scores) or satisfaction (New Scale of Sexual Satisfaction scores) over three months. However, many perceived contraception-related changes to sexual well-being. Half (51%) reported their new method had made their sex life better; 15% reported it had made their sex life worse. Sexual improvements were associated with decreased vaginal bleeding, fewer side effects, and IUD use. Negative sexual impacts were associated with physical side effects (e.g., bloating and breast tenderness), increased bleeding, and vaginal ring use. In conclusion, contraceptive users did not experience major changes in sexual functioning or satisfaction over three months, but they did report subjective sexual changes, mostly positive, due to their method.

People overwhelmingly often use contraceptives so they can have sex without experiencing pregnancy. However, most research and clinical care fails to acknowledge the sexual contexts in which people use contraception. This lack of attention to sexual pleasure can disregard the importance of sexuality in people’s overall health and well-being (Higgins & Hirsch, 2007; Landers & Kapadia, 2020); it can also undermine research and clinical practice. Many people dispense with contraceptive use due to method dissatisfaction (Pitts & Greene, 2020; Severy & Newcomer, 2005). As many as half of contraceptive users will discontinue a new method within a year (Peipert et al., 2011). The intimate link between contraception and sexuality could help explain and improve contraceptive satisfaction, which would enhance people’s ability to take advantage of the health and social benefits of contraception.

Contraceptives may have positive or negative impacts on people’s sexual experiences. They may affect people’s physical well-being via contraceptive side effects (e.g., changes in bleeding patterns or breast tenderness) or noncontraceptive benefits (e.g., improvements to dysmenorrhea). Contraception may also influence users’ psychological and emotional well-being (e.g., through disinhibition by way of feeling protected against pregnancy). All of these can influence users’ sexual well-being. Contraceptives’ effects on these sexual domains will influence overall method satisfaction and use—a phenomenon called *contraceptive sexual acceptability* (Higgins et al., 2015; Higgins & Smith, 2016).

Sexual acceptability research has documented associations between hormonal contraception and sexual function, such as arousal, lubrication, libido, and orgasm (Burrows et al., 2012; Schaffir, 2006; Welling, 2013), especially oral contraceptives’ associations with decreased libido in a small but significant subset of users (Davis & Castano, 2004; Graham et al., 1995; Schaffir, 2006). The injectable contraceptive Depo-Provera has also been associated with decreased libido among a minority of users (Gubrium, 2011; Wanyonyi et al., 2011), and vaginal rings have been associated with improvements in dyspareunia and sexual function (Caruso et al., 2014). Users of copper and levonorgestrel intrauterine devices (IUDs) have been found to report either no change or improvements in sexual function (Gorgen et al., 2009), especially by way of reduced sexual and pelvic pain and improvements in sexual desire (Bastianelli et al., 2011).

However, in addition to being stymied by study design limitations (Bancroft & Graham, 2011), the bulk of existing studies have taken a narrow, predominantly physiological approach to assessing sexual acceptability (Both et al., 2019). Physiologic phenomena such as lubrication and orgasm represent only one facet of sexual acceptability. For example, preliminary research suggests that people’s perceptions of whether their methods affect sexuality are also important components of sexual acceptability and contraceptive satisfaction and continuation (Higgins et al., 2016). Moreover, most existing studies collected data at only one point in time (Pinar et al., 2019) or, at most, after one to three months of use. Most studies assign research participants particular methods; in real-life clinical practice, patients select their own desired methods. Finally, most studies of contraceptive sexual acceptability focus only on one method at a time or compare and contrast two or three methods. Little research has examined what factors help predict positive sexual experiences across method groups—a key area of inquiry given that various contraceptive methods meet people’s needs in a variety of ways. Longitudinal research with a population of contraception-seeking clients selecting a diversity of methods will (1) strengthen understanding of the sexuality acceptability of contraceptive methods over time, (2) allow for comparisons among methods, and (3) allow documentation of factors that predict positive or negative sexual experiences across method types.

We set out to achieve these goals with a large longitudinal study of contraceptive users who selected a new method and used it for at least three months. We used a variety of measures of sexual acceptability, including measures of sexual functioning, sexual satisfaction (including psychological items), and sexual perceptions of one’s method. We were also able to document which factors, including menstrual-related changes and other side effects, were most strongly associated with positive sexual experiences of one’s method.

## Method

### Overview and Data Source

Data derive from the HER Salt Lake Contraceptive Initiative, a longitudinal cohort study nested in a quasi-experimental observational study (Sanders, Myers, et al., 2018). (HER stands for “highly effective reversible.”) The HER Salt Lake Contraceptive Initiative is registered at ClinicalTrials.Gov (NCT02734199). Survey-arm recruitment and enrollment occurred from September 2015 to March 2017. Individuals could enroll if they were between the ages of 18 and 45, spoke English or Spanish, were receiving a new contraceptive method at one of four participating family-planning health centers in Salt Lake County, Utah, and did not want to become pregnant for at least one year. Starting at the end of March 2016, all eligible clients received their method at no cost and had the ability to switch or discontinue their method at any time, for any reason, at no cost.

### Participants

The current analysis includes only those enrolled during the one year of no-cost contraception provision to eliminate the influence of cost barriers on method selection. As noted elsewhere, this group was slightly different than the all-served patient population at these four family-planning clinics during the same time period; HER Salt Lake participants were more likely to choose more highly effective methods, while all-served clients were more likely to use barrier methods or to leave the clinic with no documented contraception (Sanders, Myers, et al., 2018).

We limited this analytic sample to participants who continued, for at least three months, the same method they initiated at enrollment. We did so for two main reasons. First, most previous research on contraceptive sexual acceptability has either been cross-sectional in nature or has focused on participants who continued their method for three months. For comparison purposes, the current analyses similarly focused on continuers. Second, sexual changes due to one’s method are likely to occur in the first month of use, and certainly within three months, but are much less likely to occur suddenly after three months. Moreover, research shows that very few people stop using their method after one month only, and most discontinuation occurs after three months (Diedrich et al., 2015; O’Neil-Callahan et al., 2013). Indeed, only 10% of participants in our study stopped using their baseline method before the three-month mark.

### Procedure

We created, administered, and managed surveys through a secure web-based research electronic data capture program, REDCap, hosted at the University of Utah (Harris et al., 2009). Surveys were available in both English and Spanish and could be completed online or over the phone depending on patient preference. Participants received reminders to complete surveys via e-mail and text. The current analysis includes data collected during the baseline survey and one- and three-month follow-up surveys. Participants received gift cards to compensate for their time at both enrollment and after three months.

Following the informed-consent process with trained study staff, each participant completed the baseline survey using a computer or tablet in a private area of the family-planning health center. The enrollment survey contained a variety of sociodemographic questions and baseline health and sexuality measures. Follow-up surveys contained repeated sexuality measures as well as questions about contraceptive continuation and experiences in the past month. Participants who provided their e-mail addresses or cell phone numbers received follow-up surveys via e-mail or text, which took 5 to 10 minutes to complete. The few participants who preferred paper surveys received them by mail. The three-month retention rate was 90%.

### Study Oversight

The University of Utah Institutional Review Board (IRB) approved the “Highly Effective Reversible Contraceptive Initiative–Salt Lake (HER-SLC)” (IRB 00065794), and each participant provided informed consent to participate. The authors designed the study, and the funders had no role in the analysis or interpretation of the data, the writing of the manuscript, or the decision to submit the manuscript for publication. All authors vouched for the integrity and completeness of the data and analyses.

### Measures

Fixed sociodemographic measures came from the enrollment survey and included age, highest level of education completed, race and ethnicity information, sexual orientation, relationship status, and federal poverty level, which we calculated using 2018 cutoffs and designated as *at or below poverty level, 101% to 199% above poverty level, 200% to 299% above poverty level*, and *300% or more above poverty level*. Other fixed variables collected at baseline included the method selected at the time of enrollment, the patient’s number of typical menstrual bleeding days prior to enrollment, and study-related fixed controls, including enrollment site (that is, at which of the four clinics the participants enrolled in the study).

We collected three time-varying sexuality measures during the one- and three-month follow-up surveys, and all measures asked the participant to report on their experiences in the past four weeks. Two measures were continuous; these measures are more consistent with prior research on contraception and sexuality. An abridged, six-item version of the Female Sexual Function Index (FSFI-6; Isidori et al., 2010) measured desire, arousal, pain, lubrication, orgasm, and overall satisfaction (Rosen et al., 2000). The New Sexual Satisfaction Scale (NSSS) measured 20 items that capture physiological, psychological, partner-related, and activity-focused components of sexuality (Stulhofer et al., 2010). In both measures, larger values are associated with higher levels of sexual functioning and satisfaction, respectively. The third measure captured patients’ perceptions of contraceptive-related sexual effects: “In the last four weeks, would you say that your birth control method has …” Response options were *Improved your sex life a lot, Improved your sex life a little, Had no effect on your sex life, Made your sex life a little worse*, and *Made your sex life a lot worse* (Higgins et al., 2016).

Other time-varying measures, all of which assessed the patients’ experiences in the past four weeks, included changes in vaginal bleeding and experiences of side effects as measured by 10 items adapted from the Menstrual Symptom Questionnaire (MSQ; Chesney & Tasto, 1975; Negriff et al., 2009; Sanders, Adkins, et al., 2018). These items asked participants to report whether they had experienced any of the following side effects: headaches, bloating, breast tenderness, moodiness or irritability, acne flare-up, cramping, weight gain, weight loss, depressed mood, or constipation or diarrhea. For each side effect, response options were as follows: *Have not had in the past 30 days, Once a month, A couple of days a month, Once a week, A couple of days a week*, and *Every day*. We recoded these items into a physical side effects measure (the mean of the headaches, bloating, breast tenderness, acne flare-up, cramping, weight gain, weight loss, and constipation or diarrhea items) and mood side effects measure (the mean of the moodiness or irritability and depressed mood items). Participants also completed the World Health Organization Well-Being Index (WHO-5), a five-item measure of health and well-being (Braher et al., 2007). A widely used measure of general well-being, the WHO-5, was developed to assess subjective interpretations of one’s own physical and mental health across a number of domains.

### Analyses

**Figure 1.**
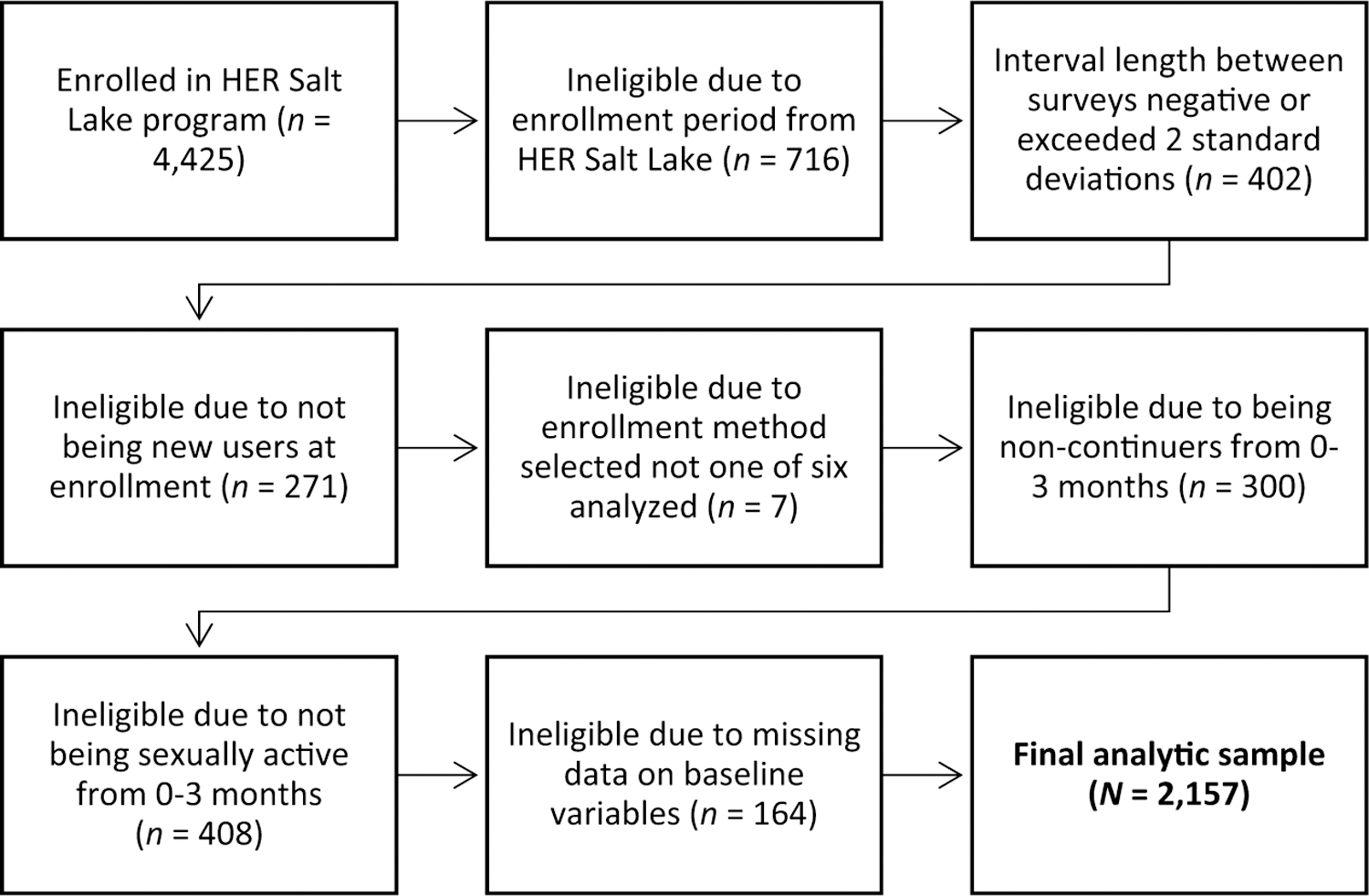
Flowchart of inclusion criteria for analytic sample.

The vast majority (99%) of our sample chose one of six methods: the contraceptive implant, copper IUD, levonorgestrel IUD, combined oral contraceptives, vaginal ring, or injectable contraception. Given that other method groups had insufficient sample sizes for method-specific analyses, they were excluded from the present analysis. (Of the original sample, 0.2% selected patches, 0.2% selected condoms, and 1.5% selected some other method, e.g., emergency contraception, fertility awareness methods, or progestin-only pills). We collapsed categories for race and ethnicity and sexual orientation due to small cell sizes. The final analytic sample size was 2,157 (Figure 1).

We conducted all analyses using Stata Version 15 (StataCorp, 2017). We first described sociodemographic characteristics by contraceptive method selected at baseline using Kruskal–Wallis tests for ordinal variables and chi-squared tests for categorical variables. Analyses then documented the perceived impact of one’s contraceptive method on one’s sex life and changes in vaginal bleeding pooled over the one- and three-month survey waves. We also assessed average changes in NSSS and FSFI-6 scores, frequency of physical and mood side effects, and values for the WHO-5 between baseline and one-month and three-month follow-up surveys. We evaluated whether these variables differed by contraceptive method using the Skillings–Mack statistic for ordinal variables and one-way analysis of variance (ANOVA) for continuous variables.

Multivariable analyses involved longitudinal, multilevel, ordered-logistic regression, with perceived impact of method on sex life as the outcome, while sociodemographic variables (age, education, race and ethnicity, poverty level, relationship status, and sexual orientation), changes in vaginal bleeding, the WHO-5, and physical and mood-related side effects were covariates. Individual fixed controls included baseline vaginal bleeding and time interval between surveys; study-related fixed controls included study period and enrollment site. The NSSS and FSFI-6 measures were not included in the multivariable models because they were outcomes, not covariates. This model accounts for nested observations within each respondent by the survey wave by correcting for clustering by respondent and by including an indicator term for the survey wave.

## Results

Analyses included the 2,157 HER Salt Lake participants who chose one of the six most-selected methods and who continued their method and completed follow-up surveys for three months. Because we collected data at baseline and at one and three months, the total number of longitudinal data points was 6,471. In terms of method profile, 31% of participants selected levonorgestrel IUDs, 23% chose implants, 17% chose pills, 14% chose copper IUDs, 10% chose injectables, and 4% chose vaginal rings (Table 1).

Table 1 presents the sample’s sociodemographic overview. In total, 62% were under the age of 25. One in three participants (35%) identified as people of color (23% Hispanic non-White, 12% non-Hispanic non-White), 43% had a high school diploma or less, and 41% reported household incomes that were at or below the federal poverty level. The majority were either cohabiting or in a committed relationship with their partner (51%) or married (12%), while 11% were single and 20% were actively dating. The majority (87%) identified as “exclusively heterosexual”; the remaining 13% claimed another sexual identity.

Our next step in the analysis was to document respondents’ sexual and side effect outcomes over three months, both for the sample overall and by individual method groups. We present categorical variables separately from continuous variables. Table 2 presents the percentages at each time point and a Skillings–Mack statistic for the ordinal variables measuring the perceived impact of contraception on sex life and changes in vaginal bleeding. Table 3 presents the means and standard deviations for the continuous variables: NSSS, FSFI-6, physical side effects, mood side effects, and the WHO-5 over time.

As illustrated in Table 2, across all method groups, participants were more likely to perceive that their contraceptive methods had positively versus negatively affected their sex lives, with approximately one-third overall reporting no sexual effects. In terms of the total sample, 51% reported their new methods had made their sex lives better in the past four weeks (26%, *Improved my sex life a lot*; 26%, *Improved my sex life a little*); 15% reported it had made their sex lives worse (2%, *Made my sex life much worse*; 12%, *Made my sex life a little worse*); and 34% reported it had not affected their sex lives. Although our objective was to document sexual outcomes among all contraceptive users, we did document differences, if any, across methods. Reports of positive sexual impacts of method were highest among copper IUD users and lowest among ring users. However, differences across methods were not statistically significant (*p* = .66).

In terms of bleeding changes over the course of three months, 38% of the total sample reported increased bleeding; 32% reported decreased bleeding; 16% reported no vaginal bleeding; and 14% reported no change. Bleeding changes differed significantly by method (*p* < .001). For example, 65% of injectable users and 54% of levonorgestrel IUD users reported either decreased or ceased bleeding compared to 19% of copper IUD users. Two-thirds (67%) of copper IUD users reported increased bleeding, compared to only 23% of pill users and 15% of ring users.

Within the total sample, we observed minimal changes in sexual functioning (FSFI-6), sexual satisfaction (NSSS), overall health and well-being (WHO-5), and physical side effects (MSQ physical) scores over time (Table 3). Scores for these variables remained virtually flat over three months. For example, the mean change in both NSSS scores and FSFI-6 scores was −0.8 out of scales ranging from 20 to 100 for the NSSS and 5 to 30 for the FSFI-6. The mean change in both physical and mood side effects was 0.1 in a scale range of 0 to 5.

Differences across method groups were nonsignificant for NSSS score deltas and physical side effect score deltas and significant for both FSFI-6 score deltas (*p* = .003) and mood side effect deltas (*p* < .001). For example, in terms of sexual functioning (FSFI-6 scores), both types of IUDs were associated with the greatest improvements in functioning (0.1 for copper IUDs and 0.07 for levonorgestrel IUDs), while injectable contraceptives and the implant were associated with the greatest decreases (−0.8 for injectables and −0.4 for implants). However, we underscore that these changes are relatively minimal given the FSFI-6 total score range of 5 to 30. Mood side effect differences across methods were more pronounced, with injectable and implant users reporting the greatest improvements (0.3 for both out of a scale of 0 to 5), a small decline among copper IUD users (−0.003), and no change among ring users.

Given the large difference in users’ perceptions of the sexual impact of their contraceptives, we then examined what factors were associated with perceived impact of method on sex life. Table 4 presents results of our longitudinal, multilevel, ordered-logistic regression model, which documents the adjusted odds of moving up one unit on the perceived-impact-of-method-on-sex-life question, from most negative to most positive (i.e., from *Has improved my sex life a little* to *Has improved my sex life a lot*). Values larger than 1 represent stronger positive associations—that is, an increased odds of reporting more positive sexual perceptions. This model clusters on each respondent. It also controls for method type, enrollment site, and time interval between surveys.

Several factors were significantly associated with more positive or more negative sexual perceptions of methods. Compared to those who reported no change in their vaginal bleeding, people who had experienced bleeding reductions had significantly higher odds of reporting more positive sexual perceptions with their methods (odds ratio [OR] = 1.43, confidence interval [CI] = 1.18–1.74; *p* < .001), while people who had experienced increased vaginal bleeding had significantly reduced odds of doing so (OR = .77, CI = .63–.94, *p* = .01). In addition, those who reported no vaginal bleeding reported increased odds of a positive impact of their methods on their sex lives (OR = 1.36, CI = 1.09–1.71, *p* = .01). Further, the greater the number of reported physical side effects, such as bloating, breast tenderness, and nausea, the lower the odds of reporting positive sexual perceptions of one’s method (OR = .77, CI = .69–.86, *p* < .001). Respondents who had higher (that is, better) underlying WHO-5 scores had significantly increased odds of reporting positive impacts of their methods on their sex lives (OR = 1.07, CI = 1.06–1.09, *p* < .001).

In terms of sociodemographics, all age groups were more likely than 18- to 19-year-olds to report more negative sexual impacts of their methods. Finally, compared to married participants, single respondents were more likely to report negative sexual impacts of their methods (OR = .74, CI = .56–.99, *p* = .04). Sociodemographic variables not significantly associated with perceived sexual impacts of methods included race and ethnicity, level of education, poverty level, and sexual orientation.

Compared to oral contraceptive users, copper IUD users had significantly increased odds of reporting more positive sexual impacts due to their methods (OR = 1.88, CI = 1.45–2.44, *p* < .001). Implant, levonorgestrel IUD, and vaginal ring users did not significantly differ from pill users in their perceptions of their methods’ sexual impacts (both *p* > .05), while injectable users were significantly less likely to report more positive perceptions of the impacts of their methods on their sex lives (OR = .74, CI = .55–.98, *p* = .04).

## Discussion

### Overview of Positive and Negative Changes Due to Contraceptive Method

Contraception is designed explicitly for use during sexual activity, but few large-scale multimethod studies exist of how contraceptives affect sexual well-being, both positively and negatively. Moreover, studies that do exist on this topic have tended to take a narrow approach to measuring contraceptive sexual acceptability, focusing primarily on sexual functioning alone. In this three-month study of more than 2,000 contraceptive clients who started and continued using a new method, we found minimal changes in sexual functioning or sexual satisfaction—at least as measured by the FSFI-6 and the NSSS. However, half of participants reported that their contraceptive methods made their sex lives better. Moreover, the proportion of those who reported that their methods made their sex lives better was at least three times greater than the proportion of those who reported it made their sex lives worse, both in the overall sample and within each method group.

Several reasons may explain why the average contraceptive client in this study reported positive sexual perceptions of their method but did not exhibit significant changes in FSFI-6 or NSSS scores. Some may argue that factors such as orgasm and lubrication are more measurable and therefore more objective or “real.” However, a large body of literature suggests that perceptions are paramount in patients’ experiences with drugs and medical devices—for example, by way of the placebo or nocebo effect (Price et al., 2008). Moreover, sexuality encompasses multiple domains, including (but not limited to) physiological functioning, sensation, and satisfaction, but also the psychological ability to “let go” during sex, partner factors, and physical issues such as vaginal bleeding, cramping, or breast tenderness, which can affect how a person feels sexually (Higgins & Hirsch, 2008). Contraception may affect all of these domains (Higgins & Smith, 2016). We argue that the field needs the development of robust multifaceted measures of sexual acceptability specific to contraception.

While we documented largely positive perceived sexual changes among contraceptive users, it deserves mention that about 15% of contraceptive clients, or approximately one in seven, reported sexual detractions due to their methods in the first three months of use. Care providers often counsel contraceptive patients to use their method for at least a few months so that potential side effects have the opportunity to stabilize. However, we underscore the importance of attending to how early sexual-related experiences with these methods may be associated with clients’ satisfaction with their methods and longer-term contraceptive use. In related analyses of these same data (Higgins et al., n.d.), those reporting sexual detractions due to their methods were significantly more likely than others to have discontinued their method by six months. In fact, their perceived sexual impact at one month more strongly predicted discontinuation at six months than more commonly established factors such as physical side effects or bleeding changes. Findings underscore how contraceptive programs and health care coverage must allow contraceptive users everywhere to switch methods as they see fit. Moreover, contraceptive clients should be encouraged to find a method that works for them sexually. Results could also inform the development of new contraceptive methods.

### Method-Specific Findings

The purpose of this analysis was to conduct a population-level examination of how contraceptives contribute to changes in sexual functioning and satisfaction or can improve or detract from individual sexual experiences. However, some method-specific differences emerged. Generally speaking, we observed the most favorable sexual outcomes among IUD and implant users and the least favorable outcomes among vaginal ring users, followed by oral contraceptive users. Both qualitative and quantitative research have documented some of the pathways through which IUDs and implants can improve people’s sexuality (Higgins et al., 2015). Research has also documented sexual detractions among ring users (Higgins & Smith, 2016) and oral contraceptive users (Graham et al., 2007; Sanders et al., 2001).

In our multivariable models, participants initiating and continuing IUDs were slightly but significantly more likely than those using other methods to report that their birth control made their sex lives better. IUDs do not suit everyone, and they should never be pushed on patients, but they do present a variety of benefits. Advantages include low maintenance, strong efficacy, and potential sexual benefits, including the possibility of increased disinhibition (Higgins et al., 2015). Not having to remember to take a pill every day or insert a new vaginal ring at the right time may also increase confidence in the method’s efficacy, which could in turn contribute to the ability to let go during sex.

However, we also wish to highlight overwhelming similarities by method, including in perceived sexual impact of method, sexual satisfaction scores, and physical side effects. People differ in what they need in a contraceptive method and what helps them feel sexually well. A variety of methods can meet people’s needs, sexually and otherwise (Higgins et al., 2020). One of the goals of the current analysis was to document the factors that help predict positive sexual experiences with contraceptives across methods. In time, findings could be used to develop better public health and clinical guidelines to help contraceptive clients find a method that works for them, sexually speaking.

Along those lines, our multivariable findings help establish some of the factors associated with perceived sexual improvements among all contraceptive continuers. Not surprisingly, participants who experienced decreased vaginal bleeding were more likely to report positive sexual changes due to their methods. Those with increased bleeding, as well as those who reported a greater number of physical side effects, such as headaches, bloating, and nausea, were understandably less likely to report sexual improvements. Instead of focusing only on contraceptive efficacy, a growing number of clinicians and researchers argue that patients may have a number of contraceptive preferences, and clients should be supported in finding methods that are most acceptable to them (Dehlendorf et al., 2013; Gomez & Clark, 2014; Lessard et al., 2012). Findings from this study further underscore the importance of doing so not only for people’s contraceptive satisfaction but also for their sexual well-being.

### Limitations

It is likely that unobservable factors influenced study participants’ sexual experiences of contraceptives and were not captured here. In complementary research with these same data, investigators are conducting factor analyses to see which domains, across a wide variety of sexual measures, may cluster together to form a more robust measure of sexual acceptability in relation to contraceptive use. Another limitation of this research is that given the study design, people chose their methods as opposed to being randomly allocated into method groups. However, we argue that these circumstances are far more comparable to real-world clinical scenarios.

Another factor that is likely to have influenced findings is that we examined only those people who continued their method for three months. In other words, this analysis purposively did *not* capture the approximately one in ten participants who discontinued their method due to sexual factors or other factors. Thus, our results may be skewed toward more positive sexual experiences than average contraceptive-seeking populations, who discontinue their method up to half of the time within the first year (Peipert et al., 2011). However, research shows that most discontinuation occurs after three months (Diedrich et al., 2015; O’Neil-Callahan et al., 2013), and only 10% of participants in our study had stopped using their baseline method before the three-month mark.

### Conclusion

This study indicates that about half of new-start contraceptive users perceive positive sexual impacts due to their method, and about one in seven report negative sexual impacts. Findings suggest a heretofore untapped benefit of contraception with which both clinicians and public health practitioners would be wise to familiarize themselves and potentially promote. Moreover, many of the factors that are associated with negative sexual perceptions, such as increased bleeding or side effects, could be ameliorated through method changes or side effect counseling and management. To promote both contraceptive satisfaction and overall sexual well-being, we should work to understand and promote sex-enhancing aspects of contraceptive methods, as well as how to best match individuals with methods they will find sexually acceptable.

## Figures and Tables

**Table 1.** Demographic characteristics, sample of family-planning clients initiating a new contraceptive method (*N* = 2,157).

	Overall (*N* = 2,157)	Implant (*N* = 503)	Copper IUD (*N* = 311)	LNg IUD (*N* = 669)	Injectable (*N* = 226)	Oral Contraceptives (*N* = 361)	Vaginal Ring (*N* = 87)	*p* Value, *H* or χ ^2^ Test Statistic, and Degrees of Freedom^a^

	%	%	%	%	%	%	%	
Age								
18–19	20.1	22.1	12.5	19.3	25.2	24.1	12.6	*p* < .001 (*H* = 60.55) *df* = 5
20–24	41.5	49.7	37.3	38.3	36.7	44.3	34.5	
25–29	22.8	19.5	28.0	24.7	19.5	19.7	31.0	
30–34	9.6	5.6	13.8	10.5	12.8	7.8	11.5	
35+	5.9	3.2	8.4	7.3	5.8	4.2	10.3	
Highest level of education completed								
Did not complete high school	4.8	6.4	2.9	4.5	4.0	4.7	6.9	*p* < .001 (*H* = 50.04) *df* = 5
High school or GED	37.8	40.2	30.9	32.7	52.2	42.1	33.3	
Some associate, vocational, technical training, or college	41.0	41.6	42.1	42.8	33.2	39.6	47.1	
Completed four-year college or higher	16.4	11.9	24.1	20.0	10.6	13.6	12.6	
Race and ethnicity								
Non-Hispanic White	65.5	60.2	68.8	70.6	57.5	64.0	72.4	*p* < .001 (χ ^2^ = 31.58) *df* = 10
Hispanic non-White	22.8	28.6	19.3	18.5	28.3	22.2	21.8	
Non-White, non-Hispanic other^b^	11.7	11.1	11.9	10.9	14.2	13.9	5.8	
Poverty category								
At or below poverty level	41.0	43.3	37.0	36.6	55.8	41.3	35.6	*p* < .001 (*H* = 35.92) *df* = 5
101%–199% above poverty level	29.0	27.6	27.7	29.6	23.5	34.4	29.9	
200%–299% above poverty level	19.1	19.1	21.2	20.6	15.0	17.5	17.2	
300% or more above poverty level	10.9	9.9	14.2	13.2	5.8	6.9	17.2	
Relationship status								
Married	12.3	12.7	12.5	13.2	11.1	11.1	10.3	*p* = .96 (χ ^2^ = 10.63) *df* = 10
Cohabiting or committed relationship	50.5	51.7	51.8	50.4	50.0	49.3	46.0	
Actively dating	19.6	17.7	19.9	18.7	19.5	22.4	25.3	
Single	11.3	11.9	9.3	12.0	13.3	9.7	11.5	
Other^c^	6.3	6.0	6.4	5.8	6.2	7.5	6.9	
Sexual orientation								
Heterosexual or mostly heterosexual	87.4	88.1	87.8	86.6	86.3	87.5	92.0	*p* = .76 (χ ^2^ = 2.59) *df* = 10
Other^d^	12.6	11.9	12.2	13.4	13.7	12.5	8.1	

*Note*. Data presented are for complete cases on baseline variables, and percentages indicate the column proportion for the specific variable. Descriptive data are presented on the nonimputed original data set. Some columns may add to slightly less than or greater than 100%; these summations are due to rounding errors; LNg = levonorgestrel; IUD = intrauterine device.

a *P* values reflect Kruskal–Wallis tests conditional on ties for ordinal variables (age, education, poverty) and χ^2^ tests for categorical variables (race and ethnicity, relationship status, and sexual orientation).

b Other category includes Asian, Native Hawaiian or Pacific Islander, American Indian or Alaska Native, African American or Black, and other.

c Other category includes divorced or separated, widowed, and other.

d Other category includes any category besides exclusively heterosexual.

**Table 2.** Contraceptive users’ experiences of methods over three months: ordinal variables, sample of family-planning clients using a new contraceptive method for three months (*N* = 3,762 observations).

	Overall (*N* = 3,762)	Implant (*N* = 900)	Copper IUD (*N* = 572)	LNg IUD (*N* = 1,198)	Injectable (*N* = 372)	Oral Contraceptives (*N* = 574)	Vaginal Ring (*N* = 146)	*p* Value and Skillings–Mack Statistic
	%	%	%	%	%	%	%	
In the last four weeks, would you say that your birth control method has:								*p* = .66 (*SM* = .09)
Made my sex life much worse	2.3	2.8	2.1	2.2	4.0	1.2	1.4	
Made my sex life a little worse	12.4	11.2	10.7	15.2	14.8	8.4	13.7	
Had no effect on my sex life	33.9	33.8	29.0	30.6	40.1	39.7	43.8	
Improved my sex life a little	25.7	24.4	27.6	27.1	19.9	27.0	24.0	
Improved my sex life a lot	25.7	27.8	30.6	25.0	21.2	23.7	17.1	
Which of the following best describes your vaginal bleeding in the last four weeks?								*p* < .001 (*SM* = 81.28)
I’ve had no vaginal bleeding	16.2	22.2	5.9	10.9	41.7	10.3	19.9	
I’ve had less bleeding than before	32.4	30.6	12.6	43.3	24.2	38.3	30.1	
I’ve had no change from before	13.5	9.9	14.9	7.9	6.2	28.4	34.9	
I’ve had more bleeding than before	38.0	37.3	66.6	37.8	28.0	23.0	15.1	

*Note*. Frequencies reflect pooled distributions of variables at one- and three-month follow-up surveys. Data presented are for complete cases in the later regression model, and percentages indicate the column proportion for the specific variable. Descriptive data are presented on the nonimputed original data set. Some columns may add to slightly less than or greater than 100%; these summations are due to rounding errors. *P* values reflect comparison of outcome by contraceptive methods using the Skillings–Mack statistic, conditional on tied rankings; LNg = levonorgestrel; IUD = intrauterine device.

**Table 3.** Changes in contraceptive users’ experiences of methods over three months: continuous variables, sample of family-planning clients using a new contraceptive method for three months (*N* = 1,881 observations).

	Overall (*N* = 1,881)	Implant (*N* = 450)	Copper IUD (*N* = 286)	LNg IUD (*N* = 599)	Injectable (*N* = 186)	Oral Contraceptives (*N* = 287)	Vaginal Ring (*N* = 73)	*p* and *F* Values and Degrees of Freedom
	*M* ±*SD*	*M* ±*SD*	*M* ±*SD*	*M* ±*SD*	*M* ±*SD*	*M* ±*SD*	*M* ±*SD*	
Change in New Sexual Satisfaction Scale (scale 20–100, where a higher score indicates better sexual satisfaction)^a^	−.8 ±8.7	−1.3 ±8.3	.06 ±8.2	−1.2 ±9.0	−.6 ±9.1	−.5 ±8.6	−.4 ±10.2	*p* = .42 (*F* = 1.00) *df* = 5
Change in FSFI-6 (scale 5–30, where a higher score indicates better sexual function)^b^	−.1 ±2.8	−.4 ±2.7	.1 ±2.4	.07 ±2.8	−.8 ±2.9	−.03 ±3.0	−.05 ±3.3	*p =* .003 (*F* = 3.64) *df* = 5
Menstrual Symptom Questionnaire: change in average experience and frequency of physical side effects (scale 0–5, where 5 represents highest frequency; symptoms include headaches, bloating, breast tenderness, acne flare-up, cramping, weight gain, weight loss, and gastrointestinal symptoms)	.1 ±.4	.1 ±.4	.1 ±.4	.09 ±.4	.2 ±.5	.08 ±.4	.08 ±.5	*p* = .16 (*F* = 1.60) *df* = 5
Menstrual Symptom Questionnaire: change in average experience and frequency of mood side effects (scale 0–5, where 5 represents highest frequency; symptoms include moodiness or irritability and depression)	.1 ±.7	.3 ±.7	−.003 ±.6	.07 ±.7	.3 ±.8	.1 ±.7	.00 ±.6	*p* < .001 (*F* = 8.80) *df* = 5
Change in WHO Well-Being Scale (scale 0–25, where a higher score indicates better overall health and well-being)	−.4 ±2.6	−.7 ±2.7	−.3 ±2.4	−.2 ±2.5	−.9 ±2.8	−.5 ±2.4	−.2 ±2.3	*p =* .01 (*F* = 2.90) *df* = 5

*Note*. Frequencies reflect average change in variables from baseline to one-month follow-up, and from one-month to three-month follow-up at the respondent level. Data presented are for complete cases in the later regression model. Descriptive data are presented on the nonimputed original data set. *F* values and *p* values compare outcome by contraceptive method using one-way analyses of variance (*df* = 5); ; LNg = levonorgestrel; IUD = intrauterine device.

a Sample size for this item was 1,595 observations.

b Sample size for this item was 1,741 observations.

**Table 4.** Ordered, multilevel, logistic regression odds ratios predicting association between covariates and family-planning clients’ reported impact of method on sex life, one to three months after enrollment (*N* = 3,762 observations).

	Adjusted Odds Ratio	Confidence Interval	Level of Significance
Baseline method			
Oral contraceptives (reference)	—	—	—
Implant	1.19	(.95–1.50)	*p* = .13
Copper IUD	**1.88**	(1.45–2.44)	***p* ≤ .001**
LNg IUD	1.15	(.92–1.42)	*p* = .22
Three-month injectable	**.74**	(.55–.98)	***p* = .04**
Vaginal ring	.82	(.59–.1.15)	*p* = .25
Bleeding changes			
I’ve had no vaginal bleeding	**1.36**	(1.09–1.71)	***p* = .01**
I’ve had less bleeding than before	**1.43**	(1.18–1.74)	***p* ≤ .001**
I’ve had no change from before (reference)	—	—	—
I’ve had more bleeding than before	**.77**	(.63–.94)	***p* = .01**
Menstrual Symptom Questionnaire: Physical side effects (scale 0–5)	**.77**	(.69–.86)	***p* ≤ .001**
Menstrual Symptom Questionnaire: Mood side effects (scale 0–5)	.99	(.92–1.06)	*p* = .75
WHO-5 Well-Being Scale (scale 0–25)	**1.07**	(1.06–1.09)	***p* ≤ .001**
Age			
18–19 (reference)	—	—	—
20–24	**.79**	(.65–.97)	***p* = .02**
25–29	**.74**	(.58–.94)	***p* = .01**
30–34	**.55**	(.40–.74)	***p* ≤ .001**
35 and older	**.61**	(.43–.86)	***p* = .01**
Highest level of education completed			
Did not complete high school (reference)	—	—	—
High school or GED	.96	(.67–1.37)	*p* = .82
Some associate, vocational, technical training, or college	.88	(.61–1.28)	*p* = .50
Completed four-year college or higher	.85	(.57–1.28)	*p* = .44
Race and ethnicity			
Non-Hispanic White (reference)	—	—	—
Hispanic non-White	.87	(.72–1.06)	*p* = .16
Non-White, non-Hispanic other	1.21	(.95–1.54)	*p* = .13
Poverty category			
At or below poverty level (reference)	—	—	—
101%–199% above poverty level	1.00	(.84–1.21)	*p* = .96
200%–299% above poverty level	1.01	(.81–1.25)	*p* = .95
300% or more above poverty level	.95	(.74–1.22)	*p* = .69
Relationship status			
Married (reference)	—	—	—
Cohabiting or committed relationship	1.14	(.90–1.45)	*p* = .28
Actively dating	1.24	(.94–1.64)	*p* = .13
Single	**.74**	(.56–.99)	***p* = .04**
Other^**†**^	1.20	(.82–1.78)	*p* = .35
Sexual orientation			
Heterosexual or mostly heterosexual (reference)	—	—	—
Other^**‡**^	1.04	(.83–1.30)	*p* = .76
Study-related fixed controls: enrollment site, study period, wave	✔		
Individual fixed controls: number of bleeding days, interval between surveys	✔		
*df*	51		
*N*	3,762		

*Note*. LNg = levonorgestrel; IUD = intrauterine device. The bold value corresponds to odds ratio that are statistically significant at the *p* < .05 level.
